# Novel Mutation Sites in the Development of Vancomycin- Intermediate Resistance in *Staphylococcus aureus*

**DOI:** 10.3389/fmicb.2016.02163

**Published:** 2017-01-10

**Authors:** Yubing Wang, Xiaoli Li, Libo Jiang, Wentao Han, Xiangming Xie, Yi Jin, Xiaoqing He, Rongling Wu

**Affiliations:** ^1^College of Biological Sciences and Biotechnology, Beijing Forestry UniversityBeijing, China; ^2^Center for Computational Biology, Beijing Forestry UniversityBeijing, China; ^3^Center for Statistical Genetics, Pennsylvania State UniversityHershey, PA, USA

**Keywords:** *Staphylococcus aureus*, vancomycin, drug-resistance, mutations, jonckheere-terpstra trend test

## Abstract

Increased use of vancomycin has led to the emergence of vancomycin-intermediate *Staphylococcus aureus* (VISA). To investigate the mechanism of VISA development, 39 methicillin-susceptible strains and 3 MRSA strains were treated with vancomycin to induce non-susceptibility, and mutations in six genes were analyzed. All the strains were treated with vancomycin *in vitro* for 60 days. MICs were determined by the agar dilution and *E*-test methods. Vancomycin was then removed to assess the stability of VISA strains and mutations. Following 60 days of vancomycin treatment *in vitro*, 29/42 VISA strains were generated. The complete sequences of *rpoB, vraS, graR, graS, walK*, and *walR* were compared with those in the parental strains. Seven missense mutations including four novel mutations (L466S in *rpoB*, R232K in *graS*, I594M in *walk*, and A111T in *walR*) were detected frequently in strains with vancomycin MIC ≥ 12 μg/mL. Jonckheere-Terpstra trend test indicated these mutations might play an important role during VISA evolution. After the vancomycin treatment, strains were passaged to vancomycin-free medium for another 60 days, and the MICs of all strains decreased. Our results suggest that *rpoB, graS, walk*, and *walR* are more important than *vraS* and *graR* in VISA development.

## Introduction

Multiple antibiotic resistant *Staphylococcus aureus* continues to be one of the most common pathogens of both hospital-associated and community-associated infections worldwide (Klevens et al., 2007; Popovich et al., 2007; Hidron et al., 2008; Kallen et al., 2010). Methicillin-resistant *S. aureus* (MRSA) infection, acquired immunodeficiency syndrome (AIDS) and viral hepatitis B are the three major infectious diseases worldwide and pose a serious threat to public health (Dantes et al., 2013). Vancomycin is the first-line antibiotic therapy for MRSA infections (Sieradzki et al., 1999; Deresinski, 2005; Moellering, 2005). However, increased use of vancomycin has led to the emergence of vancomycin-intermediate *S. aureus* (VISA) (Hiramatsu et al., 1997b). Currently, the Clinical Laboratory Standards Institute (CLSI) categorizes *S. aureus* as vancomycin susceptible (VSSA) (MIC ≤ 2 μg/mL), vancomycin intermediate resistant (4–8 μg/mL), and vancomycin resistant (VRSA) (MIC ≥ 16 μg/mL) (Patel, 2014). VISA has been reported more frequently worldwide and has aroused considerable concern (Hiramatsu et al., 1997a, 2002; Tenover and Moellering, 2007; Rishishwar et al., 2016).

VRSA emerged in 1997 due to acquisition of the *vanA* gene from vancomycin-resistant enterococci (Hiramatsu et al., 1997a; Chang et al., 2003). Previous studies indicated that spontaneous mutations play important roles in the evolution of drug resistance (Drlica, 2003; Andersson and Hughes, 2014). However, the genetic mechanism of vancomycin resistance in VISA strains has not been identified fully (Cameron et al., 2016). Several of mutations in multiple genes and gene regulation systems are needed to achieve a VISA phenotype. Several genetic alterations in two-component regulatory systems have been reported to be strongly associated with a VISA phenotype, including mutations in the *vraSR* operon (Mwangi et al., 2007), *graRS* (Howden et al., 2008b; Neoh et al., 2008; Cui et al., 2009), and *walRK* (Howden et al., 2011; Shoji et al., 2011). *VraS* may serve as a switch for the activity of the “cell wall stimulon” (Gardete et al., 2012). The *vraS* I5N mutation was found to confer heterogeneous vancomycin resistance when introduced into a vancomycin-susceptible MRSA strain (Katayama et al., 2009). The *graR* N197S mutation was suggested to convert strain Mu3 into the VISA phenotype (Neoh et al., 2008). Howden et al. confirmed that the T136I mutation in *graS* is also a key mediator of vancomycin resistance (Howden et al., 2008b). The G223D mutation in *walK* and the K208R mutation in *walR* were associated with increased vancomycin MICs (Howden et al., 2011; Shoji et al., 2011). The *rpoB* gene, which encodes an RNA polymerase subunit, also plays an important role in the evolution of VISA (Matsuo et al., 2011). The H481Y mutation in *rpoB* has been confirmed by allelic replacement experiments to increase vancomycin resistance during development of the VISA phenotype in the Mu3 (hVISA) strain (Matsuo et al., 2011). The majority of previous reports on the genetic mechanism of VISA development have focused on clinical MRSA strains (Doddangoudar et al., 2012). The development of vancomycin non-susceptibility might be affected by methicillin or other drug resistance, and so VISA development in methicillin-susceptible strains warrants investigation.

In this study, 39 methicillin-susceptible *S. aureus* and 3 MRSA strains were treated with increasing concentrations of vancomycin *in vitro* to investigate the genetic mechanism underlying development of vancomycin resistance. The genes (*rpoB, vraS, graSR*, and *walRK*) important for development of vancomycin non-susceptibility were analyzed after 60 days of vancomycin treatment and compared with those in the parental strains. Our results suggest that four novel mutation sites are important for VISA development: L466S in *rpoB*, R232K in *graS*, I594M in *walK*, and A111T in *walR*.

## Materials and methods

### Bacterial strains

Forty-two VSSA strains (numbered as S1–S42) used in this study were purchased from the China General Microbiological Culture Collection Center, China Center of Industrial Culture Collection, Agricultural Culture Collection of China, China Forestry Culture Collection Center, China Center for Type Culture Collection, China Pharmaceutical Culture Collection, National Center for Medical Culture Collections, and China Agricultural University. All strain's background were described in Table 1. Only S22 and S24 were obtained from patients, others were separated from animals or environment. Among these 42 strains, three were MRSA (S3, S34, and S37). All strains were stored at −80°C.

**Table 1:** **All strains' background**.

	**Strain ID**	**Provider**		**Strain ID**	**Provider**
S1^a^	21676^b^	CICC	S22	23656 (ATCC 25923)	CICC
S2	21600 (ATCC 27217^c^)	CICC	S23	22944	CICC
S3	01334	ACCC	S24	1.2465 (ATCC 6538)	CGMCC
S4	01340	ACCC	S25	AB 91119	CCTCC
S5	01332	ACCC	S26	10201	CICC
S6	01331	ACCC	S27	10499 (ATCC 12600)	ACCC
S7	01339	ACCC	S28	01012	ACCC
S8	10341	CFCC	S29	141405	CPCC
S9	1.8721 (ATCC 29213)	CGMCC	S30	26003	CMCC
S10	1.1697	CGMCC	S31	26112	CMCC
S11	1.1476	CGMCC	S32	01011	ACCC
S12	141396	CPCC	S33	26001	CMCC
S13	140594	CPCC	S34	141431	CPCC
S14	140575	CPCC	S35	140660	CPCC
S15	21648	CICC	S36	1.1529	CGMCC
S16	10786	CICC	S37	P1	CAU
S17	22942	CICC	S38	AB18	CAU
S18	AB 94004	CCTCC	S39	CD1	CAU
S19	AB 91093	CCTCC	S40	CD9	CAU
S20	AB 91053	CCTCC	S41	CD7	CAU
S21	23699	CICC	S42	01336	ACCC

a *Strain number*;

b *Center ID*;

c *ATCC ID*.

*CICC, China Center of Industrial Culture Collection; ACCC, Agricultural Culture Collection of China; CFCC, China Forestry Culture Collection Center; CPCC, China Pharmaceutical Culture Collection; CMCC, National Center for Medical Culture Collections; CCTCC, China Center for Type Culture Collection; CGMCC, China General Microbiological Culture Collection Center; CAU, China Agricultural University*.

### Antimicrobial susceptibility testing

The vancomycin MICs of all *S. aureus* parental strains were determined by standardized agar dilution methods, according to the CLSI guidelines (Cockerill, 2012). *E*-tests were performed using glycopeptide resistance detection strips (bioMérieux), including vancomycin, oxacillin, rifampicin, teicoplanin, according to the manufacturer's instructions. For determination of MICs, a single colony was inoculated in brain-heart infusion (BHI) broth (Oxoid, Basingstoke, UK) and incubated at 37°C. At a cell density of 0.5 McFarland units (10^8^ CFU/mL), bacteria were streaked evenly onto Mueller-Hinton agar (Oxoid, Basingstoke, UK) plates. Plates were incubated at 37°C, and the MICs were read after 18–24 h of incubation.

### *In vitro* development of vancomycin non-susceptibility

All *S. aureus* strains were incubated on BHI agar (Oxoid, Basingstoke, UK) plates with vancomycin (Sigma-Aldrich, St. Louis, MO, USA) at 50% of the initial MIC. Plates were incubated at 37°C, and the strains were passaged to fresh medium containing the same vancomycin concentration every 24 h. MICs were re-determined after 4 days of treatment using the *E*-test method. The vancomycin concentration was increased to 50% of the new MIC level of each strain. This process was repeated every 4 days for 60 days. Stability of VISA strains was then determined by passaging onto vancomycin-free agar plates every 24 h for 60 days.

### Sequence analysis and mutation detection

To identify the point mutations and amino acid changes between 60-day-treated and parental strains, the *rpoB, vraS, graS, graR, walK*, and *walR* genes of all treated and parental strains were amplified using the primers shown in Table 2. One colony of each strain was treated with lysostaphin and lysozyme, and genomic DNA was extracted using a TIANamp bacteria DNA kit (Tiangen, China) according to the manufacturer's instructions. PCR amplification using genomic DNA as the template was performed using Ex Taq DNA polymerase (Takara Shuzo Co., Ltd, Kyoto, Japan). The amplification conditions for *rpoB, graS*, and *walK* were as follows: 94°C for 3 min, 30 cycles of 94°C for 1 min, 55°C for 1 min, and 72°C for 1 min, followed by a final step at 72°C for 7 min. The amplification conditions for *vraS, graR*, and *walR* were as follows: 94°C for 3 min, 30 cycles of 94°C for 1 min, 57°C for 1 min, and 72°C for 1 min, followed by a final step at 72°C for 7 min. PCR products were purified, and their sequences were analyzed. Nucleotide and amino acid sequence comparisons of each pair of treated and parental strains were performed using DNAMAN8.0 (Lynnon Biosoft, USA). Based on the amino acid sequence alignment, the missense mutations were analyzed by Jonckheere-Terpstra trend test.

**Table 2:** **Sequences of Primers**.

**Gene**	**5′–3′ primer sequence**	**Product length (bp)**
*vraS* F	GACGTAGAGGTGATTTATCGATGAACCACT	1044
*vraS* R	TTAATCGTCATACGAATCCTCCTTATTTAA	
*graS* F	ATGAGTATGGAACTTGGCGCA	1592
*graS* R	TTCCCAGATCCAGAGGGACC	
*graR* F	GGATTAAAGATTTTCAAAGTC	675
*graR* R	GAGATTTCAAAAAATAAGCTAC	
*walR* F	ACCAGGTTGGACAGAAGACG	2000
*walR* R	TGTGCATTTACGGAGCCCTT	
*walK* F	CGCGTAGAGGCGTTGGATA	1983
*walK* R	TGGCTGTCATAGGTGTCGTT	
*rpoB* F1	GCAAGGTATGCCATCTGCAAAG	1954
*rpoB* R1	TTGCTTCGGCGATACATCCA	
*rpoB* F2	ACGTGAACGTGCTCAAATGG	2262
*rpoB* R2	ATGCCTTTGTAGCGAACACG	

The presence of *mecA* and *vanA* in all strains was detected as described previously using the following primers: *mecA* forward 5′-TGGCTATCGTGTCACAATCG-3′; reverse 5′-CTGGAACTTGTTGAGCAGAG-3′; *vanA* forward 5′-ATGAATAGAATAAAAGTTGC-3′; reverse 5′-TCACCCCTTTAACGCTAATA-3′ (Saha et al., 2008). The multilocus sequence typing (MLST) genotypes of all strains were also determined as described (Enright et al., 2000). Seven housekeeping genes of all parental strains were sequenced to obtain the sequence type (ST) of each strain.

### Statistical analysis

Based on the relationships between the MICs and time points during the vancomycin treatment, the 42 strains were grouped by hierarchical clustering (Tibshirani et al., 2001). After the *in vitro* treatment, genotypes were coded as 0, 1, or 2 for each SNP in sequenced genes. The association between each mutation site and MICs measured after 60 days vancomycin treatment was analyzed using the Jonckheere-Terpstra (JT) trend test (Jonckheere, 1954). *P* < 0.05 were considered to indicate significance. All statistical analyses were performed using the R statistical software (version 2.1).

## Results

### Development of vancomycin-intermediate resistance in *S. aureus in vitro*

**Figure 1:**
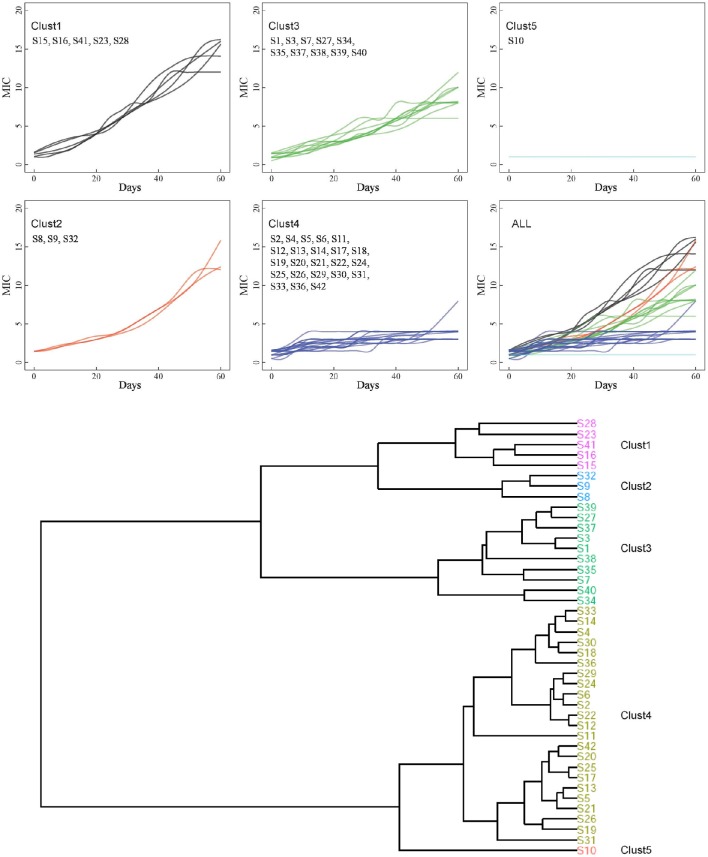
**Hierarchical clustering of 42 strains**. Based on the association between MICs and time point, the entire 42 strains were grouped into 5 clusters by hierarchical clustering.

Forty-two VSSA strains were treated with vancomycin *in vitro* for 60 days. After induction, although the MICs varied, only the MIC of the S10 strain remained unchanged compared with its parental strain. In contrast, the MICs of many strains increased significantly (Figure 1). Twenty-nine VISA strains were generated within 60 days, while 13 strains remained vancomycin susceptible (MIC < 4 μg/mL). The maximum MIC of 16 μg/mL was achieved in 4/29 strains: S8, S15, S16, and S41. The MICs of these four strains met the standard for VRSA rather than VISA strains; however, the *vanA* gene cluster, a common vancomycin-resistance determinant, was absent (data not shown). The MICs of all strains were listed in Table 3.

**Table 3:** **Amino acids mutations in VISA development**.

**Cluster**	**Strain**	**ST^a^**	**MIC^b^(μg/mL)**	***RpoB***	***vraS***	***graR***	***graS***	***walK***	***walR***
C1	S15	97	1.5/16	**L466S**^c^, H481N, T1182I	V15G	—^d^ (Q148Q)^e^	I59L, **R232K**	R222K, A468T, **I594M**	**A111T**
	S16	239	1.5/16	—(S466S, N481N)	G15V	— (Q148Q)	— (K232K)	**I594M**, (K222K, T468T)	**A111T**
	S41	9	1.0/16	**L466S**, H481N	V15G	D148Q	L26F, I59L, **R232K**	R222K, A468T, **I594M**	**A111T**
	S28	943	1.5/14	T1182I	Q126K	K17E, (Q148Q)	I59L, D97E	L265R	—
	S23	943	1.0/12	T1182I	T331I	— (Q148Q)	I193M	—	—
C2	S8	6	1.5/16	D1046V	—	— (D148D)	L59I	D266N	—
	S9	5	1.5/12	R917S	—	— (D148D)	—	G25A	—
	S32	243	1.5/12	F279L, I1182T	—	— (Q148Q)	L26F, D97E	T270S, (T468T)	—
C3	S35	8	1.5/12	**L466S**, H481N	V15G	— (Q148Q)	**R232K**	R222K, K294N, D302N, A468T, **I594M**	**A111T**
	S1	8	1.5/10	—	—	— (Q148Q)	L59I	—	P216S
	S3	239	1.5/10	T1182I, (S466S, N481N)	—	— (Q148Q)	L26F, I59L, **R232K**	— (K222K, T468T)	—
	S7	243	1.5/10	T518M	—	V136I, S207G, (Q148Q)	L59I	H385P, (T468T)	—
	S27	464	1.0/8	—	—	S79F, (Q148Q)	—	—	A111V
	S37	9	1.0/8	— (N481N)	L105F	— (D148D)	L59I, E97D	R264K, L265R, D302N	—
	S38	9	1.0/8	—	—	— (D148D)	L59I, E97D	—	—
	S39	9	1.0/8	—	G92D	— (D148D)	L26F	—	—
	S40	9	1.0/8	—	—	— (D148D)	L26F, G209S	L10F, S506Y, S591F	—
	S34	464	0.5/6	—	—	— (D148D)	—	—	—
C4	S11	96	1.0/8	D631E, T1182I	—	— (D148D)	F26L	A243T	—
	S5	239	1.5/4	Y737F, I1182T	G15V	Q148H	L59I	K540R, G560C, (T468T)	—
	S13	464	1.5/4	—	—	— (Q148Q)	—	I183K, R265L, H464Y	—
	S17	943	1.5/4	T1182I	—	— (Q148Q)	I59L, D97E	—	R119H
	S19	464	1.0/4	—	—	— (Q148Q)	—	—	F192L
	S20	464	1.0/4	—	—	— (Q148Q)	—	T471I	—
	S21	464	1.5/4	—	—	— (Q148Q)	—	V15L, R265L	—
	S25	243	1.5/4	—	—	— (Q148Q)	—	— (T468T)	—
	S26	464	1.0/4	—	G20A	— (Q148Q)	W158C	L265R, T279I	—
	S31	464	1.5/4	—	—	— (Q148Q)	—	— (T468T)	—
	S42	5	1.5/4	—	—	— (D148D)	F26L, R160H, Y223D, I224T	D302N	—
	S2	5	1.5/3	—	—	— (D148D)	—	R265L	—
	S4	243	1.5/3	—	—	— (Q148Q)	—	— (T468T)	—
	S6	243	1.5/3	P945S, I1182T	—	— (Q148Q)	I59L	— (T468T)	—
	S12	464	1.5/3	—	—	— (Q148Q)	F26L, L59I	D290E	—
	S14	464	1.0/3	G1139V, T1182I	—	— (Q148Q)	F26L	R343H	—
	S18	96	1.0/3	T1182I	—	— (D148D)	—	R265L, R298M	R222L
	S22	243	1.5/3	T1182I	—	I136V, G207S, (Q148Q)	T224I	— (T468T)	—
	S24	464	1.5/3	I1182T	—	— (Q148Q)	—	—	P216L
	S29	464	1.5/3	—	—	— (Q148Q)	—	—	—
	S30	464	0.5/3	—	—	— (Q148Q)	—	—	R107H
	S33	30	1.0/3	—	—	— (Q148Q)	F26L, Y223D	L265R, L314P	—
	S36	770	0.5/3	—	—	— (D148D)	L26F, D223Y	—	—
C5	S10	9	1.0/1	—	—	— (D148D)	L26F, D223Y, T224I	—	K208N, P216Q

a *ST, sequence type*.

b *MIC (μg/mL), MICs of parental/vancomycin treated strains*.

c *Bold indicates the novel mutations*.

d *Dash indicates amino acids had no change after 60 days vancomycin treatment*.

e *Parentheses indicates the amino acids remain unchanged*.

Based on the relationships between the MICs and time points, the 42 strains were grouped into five clusters by hierarchical clustering (Figure 1). Eight strains were found in C1 and C2 and showed the highest MICs (12–16 μg/mL). These strains exhibited rapid development of vancomycin-intermediate resistance, which was accompanied by several common mutations in sequenced genes, such as L466S and H481N in *rpoB*, R232K in *graS*, R222K, A468T, and I594M in *walK*, and A111T in *walR* (Table 3).

The third cluster comprised 10 strains. The MICs of these strains increased gradually over time, reaching 6–12 μg/mL. Twenty-three strains, including 12 VSSA strains, were grouped in C4 and exhibited slow development of vancomycin non-susceptibility, resulting in MICs of 3–8 μg/mL. Only the MIC of S11, which harbored the A243T mutation in *walK*, increased to 8 μg/mL (Table 3). Only strain S10 was grouped in C5. The MIC of S10 was unchanged after the 60 days of treatment.

The sequence type (ST) of all parental strains was shown in Table 3. Most (8/9) of the strains with MICs ≥ 12 μg/mL were assigned to different ST types.

### Antibiotic susceptibilities of all *S. aureus* strains

To determine susceptibilities to other antibiotics after 60 days of treatment, the oxacillin, rifampicin, and teicoplanin MICs of all strains were measured by the *E*-test. The MICs of several strains from each cluster are shown in Table 4. All of the 42 VSSA parental strains were initially susceptible to teicoplanin, three (S3, S34, and S37) were resistant to oxacillin, and two (S3 and S16) were resistant to rifampicin. After vancomycin treatment, the teicoplanin MICs of most strains increased, while the oxacillin and rifampicin MICs of most strains did not change. In contrast, the oxacillin MICs of five strains (S3, S15, S16, S34, and S37) decreased. Three VISA strains (S15_60_, S35_60,_ and S37_60_) became resistant to rifampicin, and the five rifampicin resistant strains carried Asn (N) in the 481st amino acid of *rpoB* (Table 3). However, the parental strains S37 and S41_60_, which also carried this Asn (N), were susceptible to rifampicin.

**Table 4:** **Antibiotic susceptibilities changes of strains**.

**Cluster**	**Strain^a^**	**MIC (μg/mL)**	**Antibiotic susceptibility**			***E*****-test MIC (**μ**g/mL)**		
			**OXA**	**RIF**	**TEI**	**OXA**	**RIF**	**TEI**
C1	S15	1.5	S	S	S	1	0.064	2
	S15_60_	16	S	R	I	0.5	>256	16
	S16	1.5	S	R	S	1	>256	4
	S16_60_	16	S	R	R	0.5	>256	16
	S41	1	S	S	S	0.5	0.064	1
	S41_60_	16	S	S	S	0.5	0.064	8
	S28	1.5	S	S	S	0.25	0.064	4
	S28_60_	14	S	S	I	0.25	0.064	16
C2	S8	1.5	S	S	S	0.5	0.064	2
	S8_60_	16	S	S	I	0.5	0.064	16
	S9	1.5	S	S	S	0.25	0.064	2
	S9_60_	12	S	S	I	0.25	0.064	16
	S32	1.5	S	S	S	0.125	0.064	2
	S32_60_	12	S	S	I	0.125	0.064	16
C3	S3	1.5	R	R	S	>256	>256	1
	S3_60_	10	R	R	S	32	>256	8
	S34	0.5	R	S	S	32	0.064	0.5
	S34_60_	6	R	S	S	8	0.064	4
	S35	1.5	S	S	S	0.25	0.064	1
	S35_60_	12	S	R	S	0.5	>256	8
	S37	1	R	S	S	>256	1	0.5
	S37_60_	8	R	R	S	4	4	8
C4	S2	1.5	S	S	S	0.125	0.064	2
	S2_60_	3	S	S	S	0.25	0.064	2
	S5	1.5	S	S	S	0.125	0.064	1
	S5_60_	4	S	S	S	0.125	0.064	2
	S11	1	S	S	S	0.5	0.064	0.064
	S11_60_	8	S	S	S	0.5	0.064	8
C5	S10	1	S	S	S	0.25	0.064	1
	S10_60_	1	S	S	S	0.25	0.064	1

*OXA, oxacillin; RIF, rifampicin; TEI, teicoplanin. S, susceptible; I, intermediate; R, resistant*.

a *No subscript represents parental strains*.

*Subscript indicates strains treated by vancomycin for 60 days*.

### Amino acid mutations involved in VISA development

The complete sequences of *rpoB, vraS, graS, graR, walK*, and *walR* were amplified from all strains. Only missense mutations were observed by amino acid sequence alignment between the 60 days' vancomycin-treated strains and parental strains. A large number of SNPs were detected, and the mutations varied among the strains. Multiple nonsynonymous mutations in the six genes are shown in Table 3.

In *rpoB*, we found 10 distinct amino acid changes in 13/29 (44.8%) VISA strains: F279L, L466S, H481N, T518M, D631E, Y737F, R917S, D1046V, and T1182I/I1182T. No amino acid substitution was found at the 466th or 481st locus in the VSSA strains treated by vancomycin for 60 days. Notably, the T1182I/I1182T mutations not only occurred frequently in VISA strains but were also found in VSSA strains. In *vraS*, seven distinct missense mutations were identified in 10/29 (34.5%) VISA strains. No nonsynonymous SNPs were found in the other VISA or VSSA strains. In the *graRS* operon, 19 distinct mutations were identified in 20/29 (69.0%) VISA strains. The L26F/F26L, I59L/L59I, and Y223D mutations were present in both VISA and VSSA strains, while the D223Y and T224I mutations were detected only in VSSA strains. Twenty-two of the 29 (75.9%) VISA strains harbored mutations in the *walRK* operon, including 23 distinct mutations in *walK* and 5 in *walR*. Among these mutations, R222K, A468T, and I594M in *walK* and A111T in *walR* occurred more frequently. Furthermore, all VSSA strains lacked R222K, I594M, and A111T but carried several other mutations.

Overall, three VISA strains (S15, S35, and S41) contained the L466S and H481N mutations in *rpoB*, accompanied by the mutations R232K in *graS*, R222K, A468T, and I594M in *walK*, and A111T in *walR* and acquired high-level vancomycin resistance (MIC ≥ 12 μg/mL). The *walK* mutation was carried most frequently by VISA strains. A previous report stated that mutations in *walK* were most frequent in 39 clinical VISA strains from various countries (Shoji et al., 2011).

**Figure 2:**
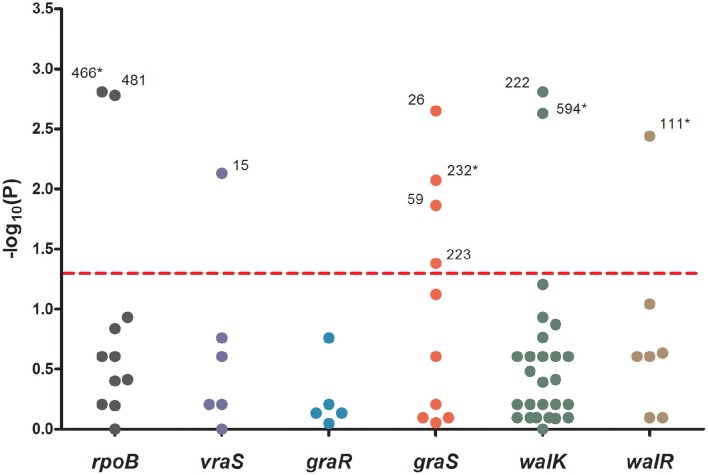
**The significance of association between each mutation sites and MICs**. Data was analyzed by Jonckheere-Terpstra (JT) trend test. The x axis shown six sequenced genes and the y axis shown −log10 of the *P* values resulting from the JT trend test. Each dot represented a mutation site, and the number beside the dot shown the amino acid position. *P* < 0.05 were considered significant. ^*^The novel mutation sites found in this study.

To analyze the associations between mutation sites and MICs, strain genotypes were coded as 0, 1, or 2 for each mutation in sequenced genes. The associations between mutations and MICs at 60 days were analyzed using the Jonckheere-Terpstra (JT) trend test. As shown in Figure 2, 10 mutations (L466S and H481N in *rpoB*, V15G in *vraS*, L26F, I59L, Y223D, and R232K in *graS*, R222K, and I594M in *walK* and A111T in *walR*) were significantly correlated with the MIC differences, including four novel mutation sites (L466S in *rpoB*, R232K in *graS*, I594M in *walK* and A111T in *walR*). These four mutation sites were only occurred in five strains with MICs ≥ 10 μg/mL and have not been reported to date. The *P* values of all mutations in *graR* were >0.05, indicating that this gene might be relatively unimportant in the process of VISA development.

### Stability of vancomycin non-susceptibility

To investigate the stability of vancomycin non-susceptibility, strains were incubated on vancomycin-free agar plates for another 60 days after the vancomycin treatment. The MICs of all strains (except S10) decreased after the removal of vancomycin, while S35 and S41 remained as VISA strains. Six genes (*rpoB, vraS, graS, graR, walK*, and *walR*) were sequenced and compared with the genes before vancomycin removal. The strains with mutations were shown in Table 5; 27 reverse mutations and 13 random mutations were detected compared with those vancomycin treated strains. Only the reverse mutation F10L in *walK* might be related to a decrease in the MIC.

**Table 5:** **Mutations after removal of vancomycin**.

**Cluster**	**Strain**	**MIC (μg/mL)^a^**	***rpoB***	***vraS***	***graR***	***graS***	***walK***	***walR***
C1	S15	3	**I1182T**^b^	—^c^	—	—	—	—
	S16	3	—	—	—	—	—	—
	S41	8	—	—	—	—	—	—
	S28	3	**I1182T**	—	—	—	**R265L**, A554G	—
	S23	3	—	—	—	—	—	—
C2	S8	2	—	—	—	—	**N266D**	—
	S9	3	—	—	—	—	—	—
	S32	2	—	—	—	**E97D**	L265R	—
C3	S35	6	—	—	—	—	**N294K**, **N302D**	—
	S1	2	—	—	—	—	—	—
	S3	2	**I1182T**	—	—	—	—	—
	S7	2	—	—	—	—	—	—
	S27	3	I1182T	—	—	—	—	—
	S37	3	—	—	—	—	**K264R**, **R265L**, **N302D**	—
	S38	2	—	—	—	—	—	—
	S39	2	T1182I	—	—	—	—	—
	S40	1	—	—	—	—	**F10L**, **Y506S**, **F591S**	—
	S34	1	—	—	—	—	—	—
C4	S11	3	—	—	—	—	—	—
	S5	3	—	—	**H148Q**, V136I, S207G	F26L	—	—
	S13	2	—	—	—	—	—	—
	S17	2	—	—	—	—	—	—
	S19	1	—	—	—	—	—	**L192F**
	S20	2	—	—	—	—	—	—
	S21	3	—	—	E15D	—	—	—
	S25	2	—	—	I136V, G207S	—	—	—
	S26	1	—	—	—	—	**R265L**	—
	S31	2	—	—	—	—	—	—
	S42	2	—	S39F	—	—	**N302D**	—
	S2	2	—	—	—	—	—	—
	S4	2	—	—	—	—	—	—
	S6	2	—	—	—	—	—	—
	S12	2	—	—	—	—	—	—
	S14	1	**I1182T**	—	—	—	—	—
	S18	2	**I1182T**	—	—	—	**L265R**	—
	S22	2	**I1182T**	—	**V136I**, **S207G**	—	—	—
	S24	2	—	—	—	—	—	—
	S29	2	—	—	—	—	—	—
	S30	1	I1182T	—	—	—	—	—
	S33	1	—	—	—	—	**P314L**	—
	S36	2	—	—	—	—	—	—
C5	S10	1	I1182T	—	—	—	—	**N208K, Q216P**

a *MIC (μg/mL), MICs after vancomycin removal for 60 days*.

b *Bold indicates reverse mutations*.

c *Dash indicates the amino acids remain unchanged*.

## Discussion

VISA strains are increasingly prevalent in the hospital setting and are a major issue in the treatment of MRSA infections. It has been suggested that the occurrence of VISA strains is relatively frequent, representing a threat to public health (Sader et al., 2009). Prolonged vancomycin exposure in patients can contribute to generation of VISA, resulting in treatment failure (Liu et al., 2011). In addition, VISA can also be generated *in vitro* from VSSA strains by exposure to vancomycin (Matsuo et al., 2011; Doddangoudar et al., 2012). Previous studies of the mechanisms of VISA development focused on clinical MRSA strains (Doddangoudar et al., 2012), while few studies have addressed VISA development in methicillin-susceptible strains. In this study, 29 (69%) VISA strains were generated from 42 *S. aureus* strains by vancomycin treatment for 60 days *in vitro*. Our results suggest that seven mutation sites are important for VISA development, including four novel mutations: L466S in *rpoB*, R232K in *graS*, I594M in *walK*, and A111T in *walR*.

Hierarchical clustering which can directly decompose the dataset into a set of disjoint clusters was performed to trace the dynamic changes in VISA development, and investigate different vancomycin non-susceptibility evolution patterns. The 42 strains were grouped into five clusters, and the MICs of nine strains (eight from C1 and C2 and one from C3) were ≥12 μg/mL (Figure 1). Among those nine strains, only S28 and S23 were assigned to the same ST type (Table 3). 38/42 parental strains was susceptible to oxacillin, rifampicin and teicoplanin. The three MRSA strains (S3, S34, and S37) were resistant to oxacillin, and 2/42 strains (S3 and S16) were resistant to rifampicin. Strains S3 and S16 developed high MICs (10–16 μg/mL) after vancomycin treatment. Three methicillin-susceptible strains—S8, S15, and S41—showed the highest MIC of 16 μg/mL. There was no significant correlation between VISA development and initial resistance to oxacillin, rifampicin or teicoplanin. The oxacillin MICs of five strains (S3, S15, S16, S34, and S37) decreased after vancomycin treatment. It has been suggested that upon acquisition of vancomycin resistance, some strains show a concomitant decrease in oxacillin resistance (Bhateja et al., 2006), and a previous study reported that mutated *graR* may impair oxacillin resistance (Neoh et al., 2008). In this study, no *graR* mutation was detected in these five strains. The H481Y/N mutation is located in the rifampin resistance-determining region, and this locus has been reported repeatedly in clinical rifampicin-resistant *S. aureus* strains (Aubry-Damon et al., 1998; O'Neill et al., 2006; Mick et al., 2010). In this study, five rifampicin-resistant strains harbored the Asn (N) mutation in the 481st amino acid of *rpoB* (Table 3). However, the parental strains S37 and S41_60_, which harbor Asn (N), were susceptible to rifampicin. In summary, no significant association was found between these mutations and antibiotic susceptibility changes.

The genetic basis for vancomycin resistance in VISA remains unclear. The *rpoB, vraSR, graSR*, and *walRK* genes have been reported to be highly associated with vancomycin resistance. In this study, the complete sequences of *rpoB, vraS, graS, graR, walK*, and *walR* were analyzed and compared with those of the susceptible parental strains. Seven mutations occurred more frequently in strains with high MICs (12–16 μg/mL), including L466S and H481N in *rpoB*, R232K in *graS*, R222K, A468T, and I594M in *walK*, and A111T in *walR* (Table 3). *In vitro* experiment and JT trend test indicated that four novel mutation sites were important for VISA development—L466S, R232K, I594M, and A111T—which were first reported in this study (Figure 2).

Alam et al. reported that *rpoB* H481 is the predominant locus associated with an increased vancomycin MIC (Alam et al., 2014). The mutations H481Y/N in *rpoB* play a dual role in rifampin and vancomycin resistance (Watanabe et al., 2011; Gao et al., 2013). Moreover, it has been suggested that the *rpoB* mutation itself, and not any other incidental genetic change caused by rifampin resistance, was responsible for the decreased vancomycin susceptibility (Matsuo et al., 2011). In this study, two mutations (L466S and H481N) were significantly associated with increased vancomycin MICs. Although the L466S mutation had not been reported previously, it might be important for the development of VISA.

In *vraS*, the 15th amino acid locus was significantly associated with MIC according to the JT trend test, but both the V15G and G15V mutations were detected in different VISA strains. Therefore, V15G/G15V seemed to be less important in VISA development.

D148Q mutation in *graR* was reported previously to be important for development of high-level resistance (Neoh et al., 2008; Doddangoudar et al., 2012). In this study, only the S41 strain contained the D148Q mutation in *graR* (MIC = 16 μg/mL), while 28/42 (66.7%) VSSA parental strains initially harbored Gln (Q) at locus 148. Therefore, we speculated that the D148Q mutation in *graR* did not significantly affect the development of vancomycin resistance. The S79F mutation is important in the development of VISA strains (Neoh et al., 2008; Shoji et al., 2011; Doddangoudar et al., 2012; Hafer et al., 2012). Among our strains, only S27 carried the S79F mutation, and exhibited a MIC of 8 μg/mL. According to the JT trend test, no mutation in *graR* and four mutations (L26F, I59L, Y223D, and R232K) in *graS* were significantly related to elevated vancomycin MICs. However, the L26F/F26L, I59L/L59I, and Y223D mutations were detected in both VISA and VSSA strains. Thus, these mutation sites were unlikely to be responsible for VISA development. The R232K mutation occurred frequently (3/9) in strains with high MICs (12–16 μg/mL) and was absent in VSSA strains. This suggests that the R232K mutation is involved in the development of vancomycin-intermediate resistance.

G223D mutation in *walK* and K208R mutation in *walR* were associated with increased vancomycin MICs (Howden et al., 2011; Shoji et al., 2011). However, these genetic changes are not observed frequently in clinical and laboratory *S. aureus* strains (Howden et al., 2008a,b; Kato et al., 2010). Shoji et al. collected 39 clinical VISA strains from various countries worldwide and then analyzed the complete sequences of *vraSR, graSR, clpP*, and *walRK*. Nine of the 39 (23%) VISA strains from four countries harbored R222K and A468T mutations in *walK*. The L10F and A243T mutations in *walK* and P216S in *walR* have been reported previously in VISA strains (Shoji et al., 2011; Hafer et al., 2012). In this study, 3/9 VISA strains with MICs ≥ 12 μg/mL harbored the R222K mutation, S3 and S16 initially carried Lys (K) at locus 222. The A468T mutation occurred in the same three VISA strains as R222K, while other ten strains carried Thr (T) including seven VISA strains initially (Table 3). Therefore, A468T seemed to be important for VISA development. According to the JT trend test, R222K, I594M in *walK* and A111T in *walR* were significantly associated with vancomycin resistance. Although the mutations I594M in *walK* and A111T in *walR* have not been reported to date, they were frequently detected (4/9) in strains with MICs ≥ 12 μg/mL. The presence of I594M and A111T in the same strain may result in development of the VISA phenotype.

Overall, seven amino acid changes—L466S, H481N, R232K, R222K, A468T, I594M, and A111T—were detected frequently in our VISA strains. Although the four novel non-synonymous mutations L466S, R232K, I594M, and A111T have not been reported to date, we speculate that these mutations may play an important role in development of VISA strains, together with the H481N, R222K, and A468T mutations, synergistically promote and maintain high-level vancomycin resistance. Several other mutations were detected in both VISA and VSSA strains or in only VSSA strains. Whether these mutations affect development of a VISA phenotype remains unknown. Notably, certain VISA strains did not harbor any important mutations within the sequenced genes. The increased MICs of these strains may be caused by mutations in other undetected/unidentified genes, such as the proteolytic regulatory gene *clp* (Shoji et al., 2011) and the accessory gene regulator *agr* (Sakoulas et al., 2002, 2005).

After removal of vancomycin for 60 days, the MICs of all strains decreased. The L10F mutation in *walK* has been reported previously in VISA strains, thus, the reverse mutation F10L in *walK* might be associated with loss of vancomycin resistance. These findings suggest that VISA development is affected by a complex gene regulatory network, although other genes or pathways might be involved in decreased MICs observed. The presence of stop codons in *vraS* and *graR* was reported to be related to loss of vancomycin non-susceptibility (Doddangoudar et al., 2012). However, no stop codon was detected in this study.

In conclusion, this study demonstrated that prolonged vancomycin exposure leads to development of vancomycin-intermediate resistance in methicillin-susceptible *S. aureus* strains. Compared with the susceptible parental strains, four novel missense mutations—L466S in *rpoB*, R232K in *graS*, I594M in *walK* and A111T in *walR—*were detected in high-level vancomycin-resistant strains. Our results also suggest that *rpoB, graS, walK* and *walR* are more important than *vraS* and *graR* in the evolution of vancomycin non-susceptibility.

## Author contributions

YJ, XH, and RW conceived and designed the experiments. YW, XL, LJ, and WH. performed the experiments. XX, YJ, XH, and RW analyzed the data, YW and YJ. wrote the manuscript. All authors reviewed the manuscript.

## Funding

This work was supported by the Fundamental Research Funds for the Central University (TD2012-03), the Special Fund for Forest Scientific Research in the Public Welfare (201404102), Natural Science Foundation of China (51108029) and a “One-Thousand Person Plan” award.

### Conflict of interest statement

The authors declare that the research was conducted in the absence of any commercial or financial relationships that could be construed as a potential conflict of interest. The reviewer SA and handling Editor declared their shared affiliation and the handling Editor states that the process nevertheless met the standards of a fair and objective review.
